# "The Hospital" Medical Book Supplement—No. XXXVIII

**Published:** 1911-01-21

**Authors:** 


### "THE HOSPITAL" MEDICAL BOOK SUPPLEMENT No. XXXVIII

CONTENTS.

PAGE

**Medicine—**
The Treatment of Syphilis by the Ehrlich-Hata Remedy ... 501
The Laws of Heredity ... 501

**Pædiatrics—**

The Care of Infants and Young Children in Health ... 502

The Care and Training of Children ... 502

The Child's Inheritance ... 502

Children: A Marchen ... 503

PAGE

**Some Health Reports—**
Annual Report on the Health of the City of Sheffield for the Year 1909 ... 503
City of Sheffield: Annual Report of the City Hospitals for the Year 1909 ... 503
Thirty-ninth Annual Report of the Ipswich Mental Hospital for the Year ending December 31, 1909 ... 504

**Miscellaneous—**
Prince Francis of Teck: a Memoir ... 504
Some Hints and Recipes for Sick-room Cookery ... 504
Essays on Duty and Discipline ... 504

### MEDICINE.

THE TREATMENT OF SYPHILIS BY THE EHRLICH-HATA REMEDY. A COMPILATION OF THE PUBLISHED OBSERVATIONS, BY DR. JOHANNES BRESLER. Second Edition, much enlarged. Translated by Dr. M. D. EDER. (London: Rebman, Limited. 1910. Price 2s. 6d.)

THE widespread interest which has been taken in the results of the arseno-benzol treatment for syphilis makes Dr. Bresler's little book of immediate value. In a convenient form will be found arranged chronologically useful summaries of the very numerous articles on the subject which have appeared of late. These largely consist of reports and addresses from German periodicals, but papers and comments from the English journals are added. The fact of a second edition being issued within a month of the first points to an appreciation of this means of getting an up-to-date bird's-eye view of the question of the efficacy of "606," and in this edition the translator, Dr. Eder, has brought the references up to the end of October. The intravenous method of injection receives careful consideration, and the technique of this, and the preparation of the solution used, is described by several workers. There is no pretence at formulating any opinions, the book is entirely a summary, and the reader is left to form his own judgment. Portraits of Ehrlich and Schaudinn are interesting embellishments of this edition.

THE LAWS OF HEREDITY. By DR. ARCHDALL REID. With index, glossary, and appendix by Prof. H. H. Turner, F.R.S. (London: Methuen. Pp. 550. Price 21s. net.)

DR. ARCHDALL REID claims to have elucidated in this bulky tome the difficulties which beset the biological student when considering "acquired" and "innate" characters. He holds that the terms are really erroneous, and that by the word "innate" we refer to those characters which have developed under the stimulus of nutriment, whereas by acquirement we imply those that have developed under the stimulus of use or injury. We must first deprecate Dr. Reid's somewhat involved style, which, on page 16, leads him to give a sentence of twenty lines, and we would point out to Dr. Reid that the greatest wisdom in the world needs to be clothed in a somewhat more attractive form. Consequently, if our criticisms may seem harsh to Dr. Reid, he has himself to thank for the unpalatable appearance of the dish. This distinction to which the author attributes so much importance would be excellent in its way did it tend to make the real problem more near solution. "All evolution," he says, "consists in germinal change." Good: but how arises this germinal change? The theoretical complexities of Professor Weissman do not bring us any light on the matter, and if we are not permitted to believe that external influences modify the germ plasm, we had better go back to the pristine simplicity of our ancestors and believe in separate creation. Darwin's natural selection does not seem to us to be a sufficiently strong basis on which to build the laws of heredity. Dr. Reid is apparently unacquainted with the writings of Hans Driesch, who in 1902, in the *Biological Zentralblatt*, xxii., said: "For intelligent people Darwinism is long since dead. All that is now said on its behalf is merely a general speech, based on the motif 'de mortuis nil nisi bonum,' and with an inner conviction of the insufficiency of the defendant." We do not propose in any way to make a detailed comparison between Hans Driesch and Dr. Archdall Reid, but we would simply point out that after conscientiously reading through Dr. Reid's book we do not see in what way he has added enlightenment on the questions and problems of heredity. Again, the name of Samuel Butler does not occur in his five hundred odd pages, but he devotes a chapter to "Conscious and Unconscious Memory" and a chapter to "Instinct, Reflex Action, and Reason." He objects to the phrase "unconscious memory," which he holds to be a contradiction in terms. Memory he defines as "the faculty for storing and recalling to *consciousness* feelings that have been there before." This distinction is rather pedantic, and the word "memory" certainly serves two purposes—to describe the process of storing, and a process of consciously drawing on that store. The position that he attributes to memory as being the last in order of evolution, following on reflex action and instinct, is perhaps Dr. Reid's most useful contribution to evolutionary psychology. But, as is so frequently the case with works which are written under the aegis of modern science, there is a superfluity of language. On page 225, for instance, Dr. Reid says: "Disease, then, is the only stringently selective agent amongst civilised men. The types it weeds out are those that are weak against disease; the survivors are those that are resistant to disease; it follows that the only racial progression, certainly the only considerable racial progression, that civilised human races undergo is one against disease." At first sight this sentence seems to give us the keynote to progression. Yet, on careful examination, all Dr. Reid has said is that those who are easily overcome by disease are overcome, and those who are not survive. But even this does not take us very far. There are so many degrees of differentiation that it is impossible to build any workable hypothesis on this very obvious fact. If one man is susceptible to smallpox, it is not inevitable that he will get it. Nor does Dr. Reid make sufficient allowance for the assistance given by medical aid to the susceptible constitution. The fact that we are more able to cope with disease is possibly an indication of progression in mental capacity. On the important question of immunity, Dr. Reid has much to say that is instructive, but, as before, his information is so clothed in words that it becomes increasingly difficult to see what he does mean. On page 263, for example, he says : "The *sufferings* of races have not altered them at all. Only the *elimination* of the unfittest has altered them." By the unfittest, Dr. Reid presumably means the more susceptible to disease. Thus we come back to the statement of page 225 without being any nearer the solution of the question of progression.

### PÆDIATRICS.

The Care of Infants and Young Children in Health. By Mildred M. Burgess, M.D. Lond. (London : H. K. Lewis. 1910. Crown 8vo. Pp. 72. Price 1s.)

We extend a cordial welcome to this unpretentious little volume, and hope that it will be helpful in the movement now so actively supported to prevent some of the wastage of infant life. The scope and arrangement of this manual follow the syllabus of the lectures on infant care and home nursing given in connection with the classes of the L.C.C. The book contains a clear and very easily comprehended exposition of the principles of feeding, and treats rationally and practically of the domestic details of the baby's life. Numerous useful hints and sound warnings are to be found in its pages. Whatever criticisms might be made, the result as a whole is a useful little guide, and were its precepts followed by the mothers of that vast multitude of children attending our State schools, much suffering and future disability would be avoided.

The Care and Training of Children. By Le Grand Kerr, M.D. (New York and London : Funk and Wagnalls Company. 1910. Pp. 233. Price 3s.)

The author has succeeded in producing a very readable little volume which, although it bears obvious traces of its American origin, contains nevertheless much of interest and profit for the English parent. The matter dealt with is chiefly confined to the mental and moral training of the child of school age, so space is not taken up by the discussion of the lengthy subjects of infant feeding and nursing. A few chapters are devoted to brief consideration of hygiene, diet, sleep, etc., but the main aim of the book is to put forward the author's views on the relations of the parent to the child. Dr. Kerr has managed in a brief space to touch on many important points, and of necessity his treatment in places becomes dogmatic and some of his testimony unsupported. An interesting chapter is the one on education at school; here we find some details about physiological fatigue periods which must be fresh to many, and a consideration of the average length of time during which the child at each age is capable of mental concentration. The author's dissertation on parental authority should be much appreciated in America, where we believe the spoilt child is rather frequently met with; the principles laid down are distinctly sound and calculated to react beneficially not only on the child but also on the parent. Although brief, the chapters on moral failings, the sex question, amusements, etc., are sufficient to indicate the lines on which intelligent parents can act best for their children. The practical helpfulness of this handy treatise to the parent who can apply the suggestive reasonings to his individual problems is likely to be very great. Many a parent might very well carefully digest the contents of this volume who would shrink from studying a larger manual.

The Child's Inheritance. By Greville Macdonald, M.D. With illustrations, appendices, and index. (London : Smith, Elder and Co. Pp. 325. 12s. 6d. net.)

In spite of the appearance of sincerity which this book presents—though, as Dr. Macdonald himself says, appearance is all we have to guide us—it is very difficult to treat such a work seriously. The author displays some familiarity with the eternal problems of life, and this familiarity has led him, perhaps, to overlook the main object for which he started. There are numerous quotations from various eminent authors, and we are glad to see how well read Dr. Macdonald must be, but we should hesitate to give his work to a young mother of ordinary intelligence in the hope that she might learn something profitable for the upbringing of her children. The book is exceedingly discursive. Starting with some encouraging remarks about reform and reformers, Dr. Macdonald states, on page 14 : "It would almost seem that the great foundational truths when uttered by ordinary men and women are so trite and self-evident that they help not at all. Indeed, many who perceive them quite clearly are too distrustful of themselves, just because of the truth's obviousness, to utter them." Dr. Macdonald has therefore avoided the obvious in a most ingenious manner. All the obvious things that might have been said on the psychological side of the child's inheritance, or even on the physical, he has omitted to say. All the obvious things that have been said by other people on art, wit, humour, imagination, he has copied out with loving care. The diagrammatic scheme of karyokinesis, which will doubtless soon be embodied in every cookery book, pictures of Norwegian peasant handicraft, illustrations of the leaf and stick mixtures—all these find a resting-place in this little encyclopædia. He has some very complimentary remarks to make about Wordsworth and Weissman as representing two schools; his appreciation of Blake is keen; his admiration for Ruskin, Tolstoi, and Behmen is meritorious; but what all this precisely has to do with the child's inheritance is hidden from us. On specialisation Dr. Macdonald has several quotations to offer, and on page 163 does at last make a statement which led us to suppose that he really had something of his own to say. "Indeed," he writes, "the education of a man's lifetime surely means nothing but the enlarging of his observations and of his power of deduction from these observations." But, unfortunately, at the top of page 165 we have some more of Richter. No doubt it gave Dr. Macdonald as much pleasure to write this book as it would have given us to read it had he not given it the title it has. As it was, we searched for something new in form relative to infantine psychology, and all we found was something quite old in matter but totally irrelevant. It is hardly a work, therefore, that can be recommended to the practitioner who is specially interested in children.

Children : A Märchen. By Hugo Salus. Translated by A. C. Caton. (Published by A. C. Caton, 22 Mount Carmel Chambers, Kensington, W. Price 1s. net.)

This daintily produced booklet is one that we can most heartily recommend to every parent. The mystery of creation was never expounded so tersely, so vividly, and yet, throughout, so delicately as Dr. Salus has done in this well-known attempt to answer his boy's question, "Father, where do the children come from?" We are acquainted (to our sorrow) with many books that endeavour to answer the question without making the reader wiser than he was before : books that are overburdened with silly sentimentalism, false symbolism, utterly out of place moral lectures, and distorted physiology. This little book, however, is not to be placed in the category of "goody-goody" works of that nature. It is an earnest, serious attempt to put before the child, in the simple language and with the appropriate similes of a folk-tale, the essentials of generation. It is a book that any parent can read aloud (except, we think, the preface) to any child over the age of six who is not mentally defective without puzzling the hearer and causing the reader to stammer and elide sentences. If it errs at all, it errs on the side of being too poetical for the average parent—not, let us be careful to add, for the average child. It is a beautiful little book, eminently worthy of being purchased and kept by the practitioner, for it is just a work of this kind that he is often asked about, and that he can safely recommend to his lay acquaintances.

### SOME HEALTH REPORTS.

Annual Report on the Health of the City of Sheffield for the Year 1909. By Harold Scurfield, M.D., C.M., Medical Officer of Health. (Sheffield : Pawson and Brailsford. 1910.)

Sheffield, with an area of 23,662 acres divided into thirteen registration sub-districts, has a population of 470,958 with a density of 19.9 persons per acre. At the census of 1901 there were 85,507 inhabited, 4,456 uninhabited, and 1,217 houses in building. The estimated increase of population for the year was 7,736, but the natural increase—*i.e.*, excess of births over deaths—was 6,198. During the year under review the number of births registered in the city was 13,296, giving a rate of 28.2 per 1,000, a figure lower by 2.6 than that for the previous year, when 14,268 births were registered. The number of marriages was 3,445, a rate of 14.6 per 1,000. This is the lowest rate ever recorded for the city. The number of deaths of residents during the year, after making corrections for deaths in public institutions, was 7,098, giving a rate of 15.07 per 1,000. This, again, is the lowest rate ever recorded in Sheffield. The zymotic death-rate for the year was 1.80 per 1,000 living. No case of small-pox was notified. Measles was the only infectious disease specially prevalent, and gave a death-rate of 0.9 per 1,000 living, as compared with 0.52, the average rate for the previous ten years. In all, 423 deaths were attributable to the disease, of which 413 occurred during the first six months of the year. The death-rate from whooping cough, 0.12 per 1,000 living, was much below the average. That from scarlet fever was 0.09 per 1,000, as compared with 0.22, the average for the preceding ten years. That from diphtheria was 0.08, the same as the year previous. The number of typhoid fever cases notified during the year was 177, the smallest figure since notification was introduced in 1889. The death-rate was, however, higher than in 1908, being 0.07 per 1,000, as compared with .056, but lower than the average for the decade 1899-1908 of 0.177. Diarrhœa was responsible for a death-rate of 0.54 per 1,000 living, and the lowest ever recorded, the average rate being 1.41. The relative absence of diarrhœa during 1909 was not peculiar to Sheffield. Tuberculous disease accounted for a death-rate of 1.49 per 1,000 living, as against the average of 1.83. Deaths from tuberculosis of the lung alone gave a rate of 1.11 per 1,000, as against the decade average of 1.24. The infantile mortality for the year was 119 per 1,000 children born, the lowest mortality ever recorded for Sheffield. These figures are probably the reflection of the relative absence of epidemic diarrhœa. Sheffield now stands seventeenth on the list of large towns as regards infantile mortality, as compared with twenty-fourth in 1908 and twenty-ninth during the five years 1904-1908. The causes of death were as follows : Chicken-pox 1, measles 422, German measles 1, scarlet fever 42, influenza 24, whooping cough 54, diphtheria 39, cerebro-spinal fever 1, pyrexia of uncertain origin 2, enteric fever 33, epidemic diarrhœa 197, diarrhœa 57, dysentery 1, malaria 1, syphilis 42, gonorrhœa 3, puerperal septicæmia 19, puerperal fever 1, lobar pneumonia 100, broncho-pneumonia 265, epidemic pneumonia 1, pneumonia (not defined) 323, erysipelas 15, septicæmia and pyæmia (not puerperal) 17, carbuncle 2, infective processes 5, pulmonary tuberculosis 524, tuberculous meningitis 88, tuberculous peritonitis 26, tuberculous enteritis 7, tabes mesenterica 12, lupus 1, tubercle of other organs 24, general tuberculosis 20, parasitic diseases 2, alcoholism 6, lead poisoning 1, rheumatic fever 23, chronic rheumatism 7, rheumatoid arthritis 6, gout 2, carcinoma 245, sarcoma 30, malignant disease (not specified) 128, rickets 31, purpura 8, anæmia 25, diabetes 42, premature birth 279, congenital defects 142, injury at birth 2, atelectasis 25, teething 34, diseases of the nervous system 429 (including convulsions 162), diseases of organs of special sense 21, diseases of the heart 602, diseases of blood-vessels 477, diseases of respiratory organs 702, diseases of digestive system 403, lymphatic diseases 15, diseases of urinary system 242, disease of generative system 15, accidents at child-birth 26, joint diseases 12, skin diseases 6, ill-defined causes 475, and violence 265, giving a grand total of 7,098, of whom 3,760 were males and 3,337 females, with one child of unknown sex and age. The report as to sanitary administration is satisfactory.

City of Sheffield : Annual Report of the City Hospitals for the Year 1909. By Egerton H. Williams, M.R.C.S., L.R.C.P., M.D. Brux., D.P.H. Cantab. ; Medical Superintendent. (Sheffield : The Independent Press, Ltd. 1910.)

At the commencement of the year there remained in the city hospitals 299 cases of infectious disease. During the twelve months 1,861 new cases were admitted and 1,863 discharged. Seventy deaths occurred, and the death-rate from all diseases, excluding consumption, was 3.6, the lowest for the past ten years. Of those who died, 18 were moribund on admission. Cases treated showed an increase of 176, as compared with the previous year. The percentages of cases admitted to the total number of notifications were scarlet fever 81.2, diphtheria 66.3, and enteric fever 76.2 per cent. The author has some interesting figures to give on the subject of dirty heads. Out of 683 girls under fourteen admitted, 111 had clean heads, 91 nits only, while 481 had nits and pediculi. Of 172 girls and women over fourteen, 64 were clean, 15 had nits, and 93 nits and pediculi. Of 760 boys under fourteen, 485 were clean, 50 had nits only, and 225 nits and pediculi. Of 160 boys and men over fourteen, 132 were clean, 16 had nits only, and 12 nits and pediculi. In 232 cases the hair had to be cut short, and in 1ʋ6 partly cut, amongst 855 females. Out of 1,659 observations made with respect to vaccination marks on admission, 455 were found to have one, 345 to have two, 215 to have three, and 449 to have four or more marks, while 195 had no marks. The author does not consider these figures at all satisfactory for a town like Sheffield, which had such a striking lesson about twenty years ago.

The author is eulogistic concerning the superiority of electric light over gas for hospital work, since it gives a concentrated light for working by, can be easily screened so as not to interfere with the sleep of other patients than the one to be attended to, and is most useful for medical examinations. He also has a few words to say regarding consumptive sanatoria and their results. These last, he maintains, are somewhat disappointing, for many cases relapse on returning to their old surroundings. He considers the Sheffield system of educating as many consumptives as possible to be far more beneficial than sending a few patients away to sanatoria in the hope of effecting a cure in individual cases. He does not deny, however, that the ideal consists in a combination of both methods, providing they are carried out thoroughly. He would like to see the erection of a sanatorium where the cure will be the practical continuation of the education on the same lines. The writer speaks favourably of the practice current at the Lodge Moor Hospital of showing patients their own sputum under the microscope. With a little explanation he has found it easy to get them to understand why they are made to shave clean, to keep their nails short, and to clean their hands immediately after soiling them with handkerchiefs or flasks. The use of linen pockets to go inside their ordinary ones no longer strikes them as a fad. The report also contains a notice regarding a fire which broke out at Lodge Moor Hospital, causing damage to the extent of £100. The chief officer of the fire brigade has nothing but praise for the workmanlike manner in which the staff dealt with the outbreak.

THIRTY-NINTH ANNUAL REPORT OF THE IPSWICH MENTAL HOSPITAL FOR THE YEAR ENDING DECEMBER 31, 1909. (Ipswich: Smith's Suitall Press. 1910.)

THIS hospital contained 128 male and 165 female patients on January 1, 1909. Thirty-six males and 38 females were admitted, 22 males and 14 females were discharged, 19 males and 19 females died, during the year, and 123 males and 170 females remained in residence on December 31, 1909. Of those discharged the percentage of recoveries on the admissions was 30.6 for men, 15.8 for women, and 22.9 for both sexes. Of those that died, lobar pneumonia accounted for 3 deaths, phthisis for 4, sarcoma for 1, cellulitis for 1, brain-softening for 1, general paralysis for 6, epilepsy for 2, cerebral meningitis for 1, heart disease for 7, cerebral hæmorrhage for 3, gangrene for 1, congestion of the lungs for 1, perityphlitis for 1, cystic kidney for 1, senile decay for 5. Of these deaths the causes were verified by post-mortem examination in 31 cases. The average age at death was 57 years. The report of the Commissioners in Lunacy is entirely satisfactory. The maintenance charge per head per week is 12s. 10d. for home patients, from 14s. to 25s. for private patients, and from 14s. to 16s. for out-county patients.

### MISCELLANEOUS.

PRINCE FRANCIS OF TECK: A MEMOIR. By REGINALD LUCAS. With three photographs. (London: A. L. Humphreys. 1910. Pp. 56. Price 1s. net.)

THIS brochure contains a vivid sketch of some phases of the life of the late Prince Francis of Teck, and gives a good idea of him as a man of keen pursuits and of versatile energy. As a soldier and as a civilian a brief account of his characteristics and his useful social works is set out by the author in a candid and lively spirit. Of the work of Prince Francis at the Middlesex Hospital there is naturally an enthusiastic appreciation. The entire profits of the sale of this book are, by the generosity of the publisher, placed at the disposal of the Prince Francis of Teck Memorial Fund of the Middlesex Hospital.

SOME HINTS AND RECIPES FOR SICK-ROOM COOKERY. By MAUD EARLE. (London: Spottiswoode and Co. 1910. Pp. 19. Price 1s.)

THE efforts of the author of this short monograph are entirely confined to the teaching of methods whereby nutritious and tempting dishes can be prepared for the invalid and convalescent patient. She makes no attempt to define what is suitable to any particular form of illness, with the exception of stating that saccharin should be used instead of sugar in preparing food for a diabetic patient. In this she is wise, for the decision as to what is good or bad for a particular case should undoubtedly rest with the medical attendant. Most of the recipes given are adapted from those in use at the N.T. School of Cookery (Mrs. Charles Clarke). The quantities given are small, with the idea of encouraging nurses to undertake the preparation of tempting little dishes, when it would be impossible to attempt the larger quantities usually given in text-books. The nurse in attendance on a convalescent will find Miss Earle's little book of great utility when desiring to introduce a change in the somewhat monotonous diet allowed such patients, for though the substance of the meal remains the same, it is possible with a little trouble to so change its appearance as to remove the feeling of monotony and stimulate a jaded appetite.

ESSAYS ON DUTY AND DISCIPLINE. A Series of Papers on the Training of Children in relation to Social and National Welfare. Vols. I., II., and III. (London: Cassell and Co., Ltd. 1910. Each volume containing ten essays. Price per volume, 1s. paper; 2s. 6d. cloth; 5s. leather net.)

THE universal favour with which the publication of this series has been met by men and women of all grades of society and of all schools of thought makes it almost superfluous for us to add our voice to the chorus of approval which has already hailed the publishers' enterprise. It is, however, with the hope that this notice may catch the eye of a few members of our profession who have not read these essays that we state that they are in every way worthy of study, and more especially perhaps by those who still have need for a nursery in their homes. It does not need the brain of a genius to perceive that there is about us in all countries a spirit of unrest, indiscipline, and rebellion against all recognised authority, and that this spirit is not confined to any one class of society. We are inclined to believe that this evil is to a large extent the product of education upon wrong lines. In abandoning the advice of Solomon we have indeed spoiled the child, and the child is father to the man. If these essays can do anything to mitigate the evil in the coming generation they will not have been published in vain.

